# Drug Reaction Causing Acute Tubulointerstitial Nephritis: A Molecular Mistake

**DOI:** 10.7759/cureus.117286

**Published:** 2026-10-01

**Authors:** Isabel Moreira, Mariana Gomes, Neuza Soares, Jorge S Almeida

**Affiliations:** 1 Internal Medicine, Hospital de São João, Porto, PRT; 2 Infectious Disease, Hospital de São João, Porto, PRT; 3 Internal Medicine, Centro Hospitalar Universitário de São João, Porto, PRT; 4 Medicine, Faculdade de Medicina da Universidade do Porto, Porto, PRT

**Keywords:** acute tubulointerstitial nephritis, checkpoint inhibitors, pembrolizumab, polymedication, proton pump inhibitor

## Abstract

Kidney diseases can affect different kidney structures, namely, the glomerulus, the tubules, and the interstitium. Acute tubulointerstitial nephritis (ATIN) affects both the tubules and the interstitium, initially through epithelial damage that can progress to interstitial fibrosis and tubular atrophy. There are different mechanisms that can produce ATIN, and most of them involve a renal immune response.

The case involves a 53-year-old woman with breast cancer undergoing chemotherapy and pembrolizumab who was concurrently receiving a proton pump inhibitor and antibiotic therapy for an *Escherichia coli *urinary tract infection. She presented with persistent acute renal injury. Initial laboratory and imaging investigations did not reveal significant abnormalities. A kidney biopsy was performed, which demonstrated histopathological findings consistent with ATIN. Corticosteroid therapy was initiated, resulting in gradual improvement of renal function.

This case aims to demonstrate the complexity of managing polymedicated patients and also shows the importance of timely diagnosis and prompt removal of the etiological agent.

## Introduction

Acute tubulointerstitial nephritis (ATIN) can be caused by infections, autoimmune diseases, and other conditions [1].Drug-induced acute interstitial nephritis (AIN) is an important cause, and it is also common with nonsteroidal anti-inflammatory drugs, antibiotics, and proton pump inhibitor (PPI) use [2].

However, new drugs have been described as causing ATIN. The use of checkpoint inhibitors aims to reactivate T cells, which were inhibited by tumor cells and may also disinhibit autoreactive T cells, leading to immune-related adverse events in the kidney [1].

The case presented involves a woman receiving treatment with chemotherapeutic agents, immune checkpoint inhibitors, and PPIs, who developed acute kidney injury (AKI) and a urinary tract infection. Antibiotic therapy was therefore initiated, resulting in an improvement in inflammatory markers; however, renal function continued to deteriorate. Given the persistent worsening of kidney function, AIN was suspected and subsequently confirmed by renal biopsy.

Managing patients receiving multiple potentially nephrotoxic or immune-mediated therapies is challenging for physicians because it becomes difficult to assess drug causality.

In the present case, the concomitant exposure to multiple potentially nephrotoxic agents complicates the attribution of AKI to a specific offending drug, thereby underscoring the diagnostic complexity of the case.

## Case presentation

A 53-year-old woman presented with left breast cancer, triple-negative, under neoadjuvant treatment with paclitaxel/carboplatin and pembrolizumab for the past two months, and gastroesophageal reflux disease under PPI for the past three months. She was moved to the emergency department, sent from the oncology department due to a week of rash, asthenia, and fever. Laboratory tests showed AKI (serum creatinine increased from a baseline level of 0.59 mg/dL to 2.15 mg/dL), elevated inflammatory markers, urinalysis with elevated leukocytes and proteinuria, without erythrocyturia, white blood cell casts, or eosinophiluria, in addition to *Escherichia coli *on urine culture. She had elevated hepatic parameters, which were not present in previous laboratory tests. The results of the laboratory tests are presented in Table 1. 

**Table 1:** Laboratory tests in the Emergency Department RBC: Red Blood Cells; WBC: White Blood Cells; uL: Microliter; g: Gram; dL: Deciliter; %: Percent; mg: Milligram; mEq: Milliequivalent; L: Liter; U: Unit; ASAT/GOT: Aspartate Aminotransferase; ALAT/GPT: Alanine Transaminase; GGT: Gamma-Glutamyl Transferase; ALP: Alkaline Phosphatase; CRP: C-Reactive Protein; ACR: Albumin-to-Creatinine Ratio

Parameter	Patient Value	Reference Range	Unit
Hematology			
RBC	4.12	4-5	10^12^/uL
Hemoglobin	12.0	12-16	g/dL
WBC	↑ 21.39	4-11	10^9^/uL
Neutrophils	↑ 85.6	53.8-69.8	%
Eosinophils	0.7	0.6-4.6	%
Lymphocytes	23.4	22.6-36.6	%
Platelets	216	150-400	10^9^/uL
Biochemistry			
Urea (peak)	35	10-50	mg/dL
Creatinine (peak)	↑ 2.15	0.51-0.95	mg/dL
Sodium	136	135-147	mEq/L
Potassium	5.0	3.5-5.0	mEq/L
Chloride	101	101-109	mEq/L
Inflammatory & cardiac markers			
CRP (range)	↑ 93.4	<3.0	mg/L
Urinalysis			
WBC	↑ 196.7	<30	/uL
RBC	3.7	<27	/uL
ACR	↑ 80	<30	mg/g
Total casts	0	<2.5	/uL
Hyaline casts	0	<2.5	/uL
Pathological casts	0	<2.5	/uL

At the time, renovesical ultrasound revealed no significant findings. The diagnoses were ATIN and urinary tract infection. Amoxicillin/clavulanic acid and corticosteroid therapy (prednisolone 1 mg/kg/day) were initiated immediately in the emergency department and continued at home for a total of seven days. Eight days following the initiation of antibiotic and corticosteroid therapy, the patient returned to the emergency department. She was asymptomatic, with improvement in leukocytosis and elevated inflammatory markers and without eosinophilia, but still experienced impaired renal function (Table 2).

**Table 2:** Laboratory tests after eight days RBC: Red Blood Cells; WBC: White Blood Cells; uL: Microliter; g: Gram; dL: Deciliter; %: Percent; mg: Milligram; mEq: Milliequivalent; L: Liter; CRP: C-Reactive Protein

Parameter	Patient Value	Reference Range	Unit
Hematology			
RBC	4.20	4-5	10^12^/uL
Hemoglobin	12.3	12-16	g/dL
WBC	↑ 11.30	4-11	10^9^/uL
Neutrophils	50.2	53.8-69.8	%
Eosinophils	0	0.6-4.6	%
Lymphocytes	23.1	22.6-36.6	%
Platelets	193	150-400	10^9^/uL
Biochemistry			
Urea (peak)	↑ 105	10-50	mg/dL
Creatinine (peak)	↑ 2.20	0.51-0.95	mg/dL
Sodium	139	135-147	mEq/L
Potassium	4.3	3.5-5.0	mEq/L
Chloride	101	101-109	mEq/L
Inflammatory & cardiac markers			
CRP (range)	↑ 6.8	<3.0	mg/L

Due to worsening renal function, the patient was admitted for further investigation. To address the clinical hypothesis of acute iatrogenic or autoimmune tubulointerstitial nephritis, further testing was carried out.

The laboratory tests, on the first day of hospitalization, for autoimmune diseases came back negative. The sediment rate was elevated (108 mm/hour) without immunoglobulins, kappa/lambda chain ratio, or complement elevation; protein electrophoresis without a monoclonal peak and anti-glomerular basement membrane antibodies were negative.

The kidney biopsy performed on the second day of hospitalization showed 10 glomeruli, one with global sclerosis and the other with no significant findings. The interstitium had moderate lymphocytic inflammatory infiltrate with rare plasma cells and tubulitis. There were no granulomas. Routine immunofluorescence was unremarkable. Together, these findings were compatible with tubulointerstitial nephritis. The images obtained from the kidney biopsy can be observed in Figure 1 and Figure 2.

**Figure 1:**
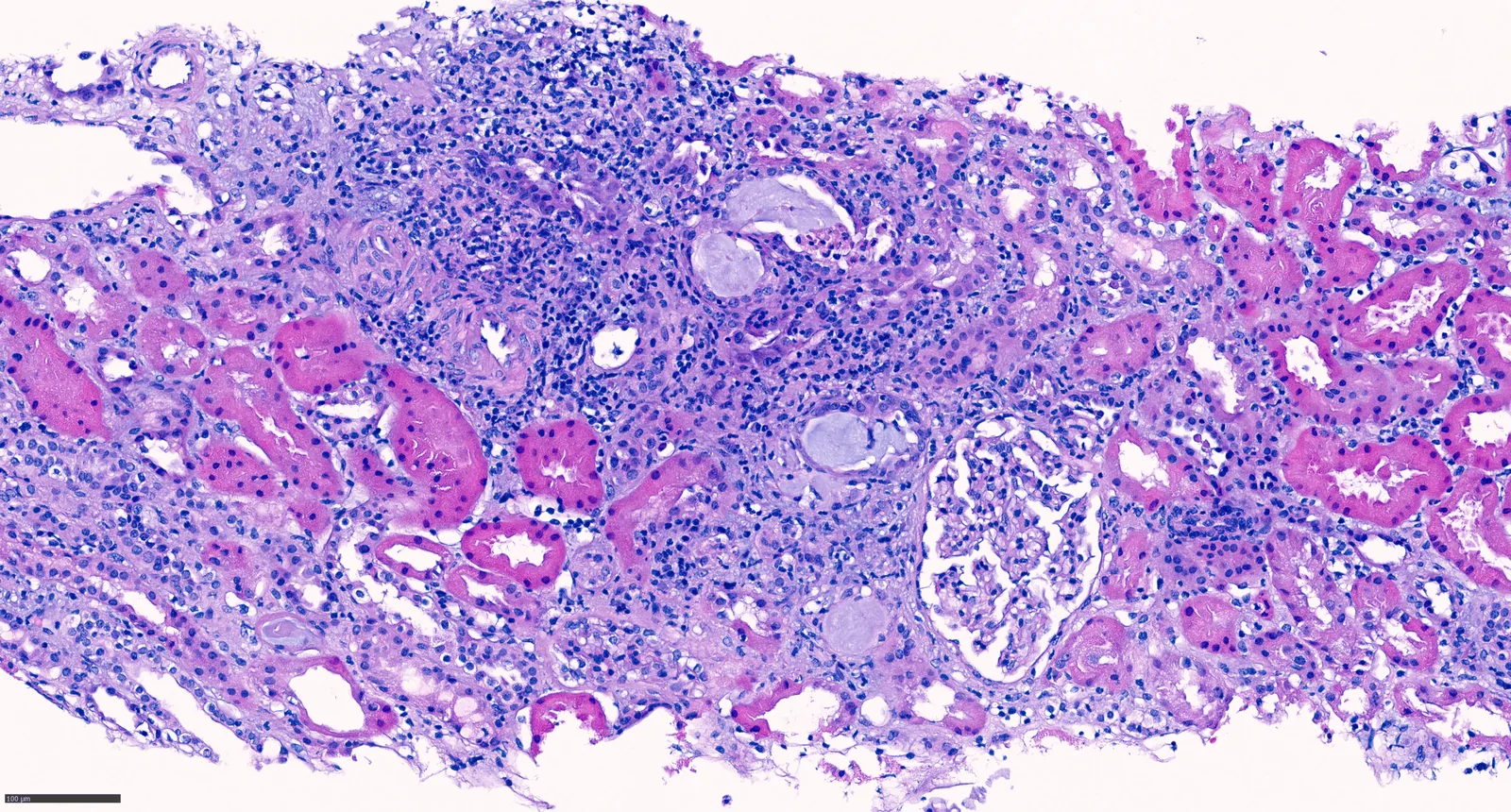
Kidney biopsy with moderate inflammatory infiltrate in the interstitium (hematoxylin-eosin stain, at 100x magnification)

**Figure 2:**
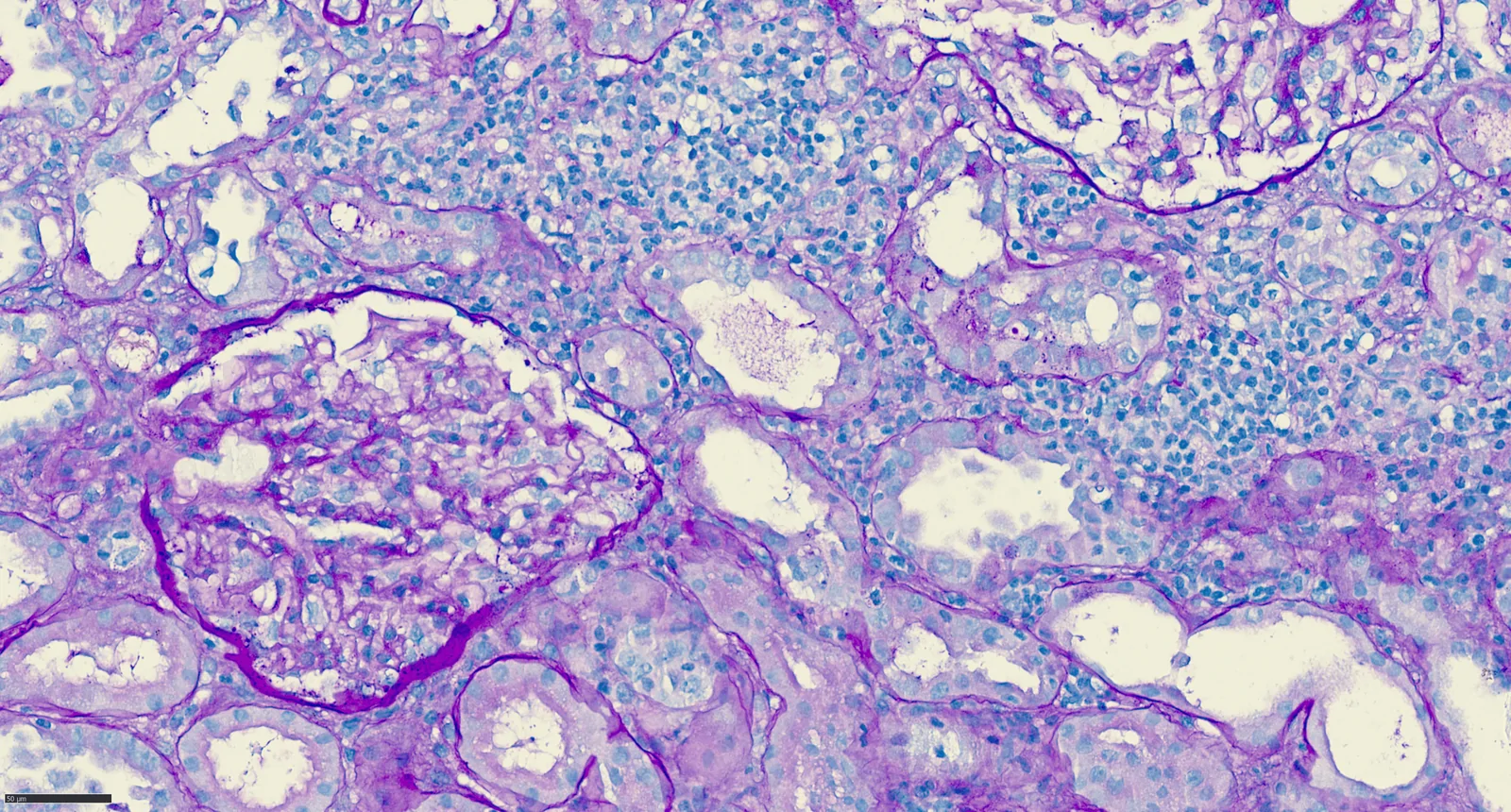
Kidney biopsy with some inflammatory cells with permeation of tubular epithelium - tubulitis (PAS stain, at 200x magnification) PAS: Periodic Acid-Schiff

A diagnosis of ATIN was made due to probable cross-reaction of pembrolizumab with the PPI and amoxicillin/clavulanic acid, and with concomitant hepatic and dermatologic toxicity.

Due to the effects of the PPI, it was discontinued, and famotidine was initiated. She started prednisolone 60 mg with renal function improvement (creatinine 1.48 mg/dL).

Three weeks after discharge from hospitalization, she experienced recovery of renal function, maintaining elevated serum creatinine compared to baseline. Six weeks later, she presented with a plateau of renal function (creatinine 1.00 mg/dL).

Concerning breast cancer treatment, immunotherapy with pembrolizumab was discontinued. Five months later, she underwent a bifocal enlarged lumpectomy of the left breast and initiated adjuvant radiotherapy.

## Discussion

The tubules and the interstitium are responsible for many renal functions, particularly glomerular filtration, hydro-electrolytic and acid-base balance, metabolic excretion, and hormone activity [3].For this reason, when these structures are affected, severe changes in systemic stability may occur, with subsequent acute or chronic disease development.

Concerning ATIN, the physiopathological explanation is an immunological response of the kidney. There is a type IV hypersensitivity reaction mediated by T cells [3,4].

The immune and inflammatory processes can be definitively diagnosed with a renal biopsy that shows interstitial edema, infiltration with T cells, eosinophils, and sometimes lymphocytes forming non-necrotizing granulomas. Tubulitis may also be observed [1,3].

Although studies have shown that beta-lactam antibiotics, PPIs, and nonsteroidal anti-inflammatory drugs are relevant causes of ATIN, the use of immunotherapies is increasing, and their adverse effects are becoming more robust [3,5].

The case presented describes a probable cross-reaction between pembrolizumab, PPIs, and a beta-lactam antibiotic taken. Although each of the drugs previously mentioned has been associated with ATIN, it is difficult to identify the true causative agent in polymedicated patients. The use of a formal causality assessment tool (e.g., the Liverpool Causality Assessment Tool or the Naranjo algorithm) could have strengthened the attribution of the adverse event [5].

It was imperative to suspend immunotherapy with pembrolizumab and review the medication for gastroesophageal reflux disease to remove the trigger of the renal insult. This case highlights the importance of constantly reassessing patient treatments to avoid drug interactions and their complications.

Analyzing this case allows us to draw important conclusions about the management of complex patients. Firstly, this case allows us to reflect on the importance of diagnostic suspicion in patients taking multiple medications, which may have immune-mediated responses and drug interactions. Clinical manifestations are nonspecific and can include fever (most common), skin rash, and arthralgia [3,4].Abdominal pain can arise from distension of the renal capsule due to infiltration of inflammatory cells [6]. This clinical presentation was evident in the case presented.

Secondly, this case emphasizes the importance of the timely approach to ATIN. Although some patients recover kidney function, some may progress to chronic kidney disease, and around 20 to 30% will require chronic dialysis [2,7].Fortunately, in our case, kidney damage gradually improved until the patient reached her baseline creatinine level.

Triple-negative breast cancer treatment, cT1c-4 or N+, includes neoadjuvant therapy followed by surgery and, in certain cases, radiotherapy. Neoadjuvant therapy allows downsizing of a large unifocal primary tumor, preparing for breast-conserving surgery. This therapy includes chemotherapy and immunotherapy (e.g., pembrolizumab) [8].

The combination of chemotherapy and immunotherapy aims to target residual disease and prevent recurrence through the eradication of micrometastasis and prolonged remission. Studies also showed that this combination had a positive impact on surveillance, with pathological complete response at the time of surgery [9,10].The importance of pembrolizumab as an adjuvant treatment has also been reported [10].

Considering the importance of pembrolizumab in the treatment of this patient, the decision to suspend this drug had an impact on the recovery of renal function but may have altered the course of the cancer.

The patient completed chemotherapy cycles and underwent a lumpectomy. She started adjuvant radiotherapy without the association of checkpoint inhibitors, which may affect survival.

## Conclusions

This case highlights the importance of a multidisciplinary approach, including internal medicine, nephrology, and oncology departments. It illustrates the physician’s role in managing polypharmacy and underscores the need for careful medication review to optimize patient outcomes. A comprehensive review of the patient’s oncological and non-oncological therapies was essential to prevent further complications.

This case also highlights the value of assessment scales in improving diagnostic accuracy and guiding treatment, while suggesting possible cross-reactivity between the drugs involved in AIN, although the causative agent remained unidentified.
